# Effects of Different TiO_2_/CNT Coatings of PVDF Membranes on the Filtration of Oil-Contaminated Wastewaters

**DOI:** 10.3390/membranes13100812

**Published:** 2023-09-27

**Authors:** Ákos Ferenc Fazekas, Tamás Gyulavári, Zsolt Pap, Attila Bodor, Krisztián Laczi, Katalin Perei, Erzsébet Illés, Zsuzsanna László, Gábor Veréb

**Affiliations:** 1 Department of Biosystem Engineering, Faculty of Engineering, University of Szeged, Moszkvai Blvd. 9., H-6725 Szeged, Hungary; 2 Doctoral School of Environmental Sciences, University of Szeged, Rerrich Béla Sq. 1, H-6720 Szeged, Hungary; 3 Department of Applied and Environmental Chemistry, Institute of Chemistry, University of Szeged, Rerrich Béla Sq. 1, H-6720 Szeged, Hungary; 4 Centre of Nanostructured Materials and Bio-Nano Interfaces, Institute for Interdisciplinary, Research on Bio-Nano-Sciences, Treboniu Laurian 42, RO-400271 Cluj-Napoca, Romania; 5 STAR-UBB Institute, Mihail Kogălniceanu 1, RO-400084 Cluj-Napoca, Romania; 6 Department of Biotechnology, Institute of Biology, University of Szeged, Közép Alley 52, H-6726 Szeged, Hungary; 7 Institute of Biophysics, Biological Research Centre, Hungarian Research Network, Temesvári Blvd. 62, H-6726 Szeged, Hungary; 8 Department of Food Engineering, Faculty of Engineering, University of Szeged, Mars Sq. 7, H-6724 Szeged, Hungary

**Keywords:** membrane filtration, oil emulsions, PVDF, TiO_2_, functionalized CNTs, hydrophilicity, zeta potential, coalescence

## Abstract

Six different TiO_2_/CNT nanocomposite-coated polyvinylidene-fluoride (PVDF) microfilter membranes (including –OH or/and –COOH functionalized CNTs) were evaluated in terms of their performance in filtering oil-in-water emulsions. In the early stages of filtration, until reaching a volume reduction ratio (VRR) of ~1.5, the membranes coated with functionalized CNT-containing composites provided significantly higher fluxes than the non-functionalized ones, proving the beneficial effect of the surface modifications of the CNTs. Additionally, until the end of the filtration experiments (VRR = 5), notable flux enhancements were achieved with both TiO_2_ (~50%) and TiO_2_/CNT-coated membranes (up to ~300%), compared to the uncoated membrane. The irreversible filtration resistances of the membranes indicated that both the hydrophilicity and surface charge (zeta potential) played a crucial role in membrane fouling. However, a sharp and significant flux decrease (~90% flux reduction ratio) was observed for all membranes until reaching a VRR of 1.1–1.8, which could be attributed to the chemical composition of the oil. Gas chromatography measurements revealed a lack of hydrocarbon derivatives with polar molecular fractions (which can act as natural emulsifiers), resulting in significant coalescent ability (and less stable emulsion). Therefore, this led to a more compact cake layer formation on the surface of the membranes (compared to a previous study). It was also demonstrated that all membranes had excellent purification efficiency (97–99.8%) regarding the turbidity, but the effectiveness of the chemical oxygen demand reduction was slightly lower, ranging from 93.7% to 98%.

## 1. Introduction

The proportion of oil-contaminated waters is significant in global wastewater production due to various sectors, such as oil refineries, natural gas processing plants, and in the petrochemical, pharmaceutical, food, and metallurgical industries [1,2,3]. Even small amounts of oil released into the environment can cause considerable harm (reduced oxygen transport, possible suffocation, and poisoning, etc.). Moreover, oils contain various organic compounds that can be detrimental to human health. For instance, polycyclic aromatic hydrocarbons (PAHs) can cause chronic illnesses, such as osteoporosis and cancer [3,4,5]. Therefore, it is essential to purify oil-contaminated water to protect both the ecosystem and human health. Free (d > 150 µm) and dispersed (20–150 µm) oil droplets can be removed by traditional methods, like skimming and flotation (which can be supported by chemical destabilization), but the effective elimination of emulsified (5–20 µm) and dissolved (<5 µm) oils requires advanced methods, such as the use of hydrophobic adsorbents [6], advanced oxidation processes [7], or membrane filtration [8,9].

Membrane filtration is a chemical-free, easy-to-use, and effective method to eliminate oily contaminations, including micro- and nanosized oil droplets. However, the fouling of membranes by hydrophobic contaminants is a major obstacle to their use since even a thin hydrophobic layer can significantly reduce the water flux. Membrane surfaces can be hydrophilized to reduce the hydrophobic–hydrophobic (membrane–pollutant) interactions and, therefore, to moderate the cake layer formation on the surface and the fouling of the pores. Various in situ and ex situ techniques for developing membrane materials have been explored to improve the filtration performance of membranes. In the former case, additives can be loaded into dope or coagulation solutions to incorporate them into membrane matrices [10,11,12]. In the latter case, chemical or physical surface coatings can be considered [13,14,15,16]. Among other materials (like graphene oxide [17], sodium alginate hydrogel [18], calcium carbonate [19], etc.), metal and metal-oxide nanoparticles (such as Ag, Cu, Al_2_O_3_, MgO, ZnO, SiO_2_, ZrO_2_, Fe_2_O_3_, and TiO_2_ [20,21]) are also widely used as additives in the field of membrane modification to improve the filtration performance by altering the structural and physicochemical properties of the membranes, such as hydrophilicity [17,18,20,21,22,23], porosity [12], electrical charge [15], and chemical, thermal, and mechanical stability [10].

One of the most widely used nanoparticles for membrane modification is titanium dioxide (TiO_2_) because of its advantageous properties, such as hydrophilicity, high chemical stability, low cost, availability, and, most importantly, its high photocatalytic activity, which allow the development of self-cleaning membranes. Several studies have reported positive results in the design of nanocomposite membranes using carbon nanotubes (CNTs) in addition to TiO_2_ particles. CNTs have become the focus of interest in several fields because of their beneficial properties, such as high flexibility, low mass density, porous construction, high specific surface area, and excellent conductivity [24,25]. TiO_2_/CNT composite coatings have also demonstrated beneficial effects during the membrane filtration of oil emulsions, providing enhanced photocatalytic activity and reduced fouling [23,26,27,28,29,30]. Veréb et al. [26] used such a nanocomposite (containing 99% TiO_2_ and 1% CNT) for coating a PVDF ultrafiltration membrane, which was used for the filtration of oil emulsions containing 100 ppm crude oil, and significantly higher flux and lower total filtration resistance were achieved with the modified membrane. In another study, PVDF-TiO_2_/CNT_1%_ composite membranes provided an enhanced flux and flux recovery ratio during the membrane filtration of real oil field-produced water [27]. In another recent study [23], the CNT content of TiO_2_/CNT-composite-coated PVDF ultrafilter membranes was optimized for the membrane filtration of a crude oil emulsion, and 2 wt.% CNT content proved to be the most beneficial. This composition resulted in four times higher flux (510 L·m^−2^·h^−1^) compared to the one achieved with the uncoated membrane (130 L·m^−2^·h^−1^) at 0.1 MPa transmembrane pressure. Moreover, the TiO_2_/CNT_2%_-coated membrane provided almost seven times higher flux (compared to the uncoated membrane) at 0.3 MPa transmembrane pressure (1340 L·m^−2^·h^−1^) and excellent purification efficiencies (while the purification efficiency decreased significantly for the uncoated membrane at 0.3 MPa transmembrane pressure). The beneficial effects were associated with the negative surface zeta potential of the CNT, resulting in a significant electrostatic repulsive force between the droplets and the membrane surface, reducing the adherence of the oil droplets to the membrane surface. Similarly, Esfahani and colleagues also reported increased antifouling properties caused by a more negative membrane surface conveyed by the presence of CNTs (during the filtration of a bovine serum albumin solution) [31].

Based on the findings presented above, TiO_2_/CNT nanocomposite-modified membranes containing –OH and/or –COOH-functionalized CNTs can be promising for advanced separation of oil-in-water emulsions due to the potentially more negative and polar surfaces. Moslehyani et al. [32] already applied oxidized CNTs and achieved lower fouling with surface-modified CNT-containing membranes during the separation of oil-in-water emulsions. The aim of the present study was also to investigate the possible beneficial effects of CNT functionalization by increasing the repulsive forces between the oil droplets and the TiO_2_/fCNT-composite-modified membrane surfaces and to achieve higher fluxes and lower filtration resistances during the filtration of oil emulsions. However, unexpected results—originating mainly from the change in the crude oil used—widened our investigations to further directions, which gave information about the effects of the composition of the oil emulsion.

## 2. Materials and Methods

### 2.1. Modification of the Membranes

Flat sheet PVDF microfilter (0.2 µm) membranes (New Logic Research Inc., Minden, LA, USA) were coated with the nanocomposites and used as a reference. Six different multi-walled carbon nanotubes (MWCNTs) with a length of 0.5–2 µm and a diameter of <8 nm were used for the membrane modifications in all cases. The TiO_2_/CNT nanocomposites were prepared using 98 wt.% TiO_2_ and 2 wt.% CNT, as this composition proved to be the most beneficial in an earlier study [23]. The characteristics of MWCNTs and the names assigned to them are listed in Table 1.

CNT_a_-OH and CNT_a_-COOH MWCNTs (Nanografi Nanotechnology AS, Ankara, Turkey) were commercially functionalized with the hydroxyl and carboxyl groups, while CNT_b_-HNO_3_ and CNT_b_-HNO_3_/H_2_SO_4_ samples (Alfa Aesar, Waltham, MA, USA) were modified by 15 M HNO_3_ and 10 M HNO_3_/H_2_SO_4_ acid treatments of CNT_b_, respectively. The membranes were coated with the TiO_2_/CNT nanocomposites using a physical deposition method [33]. Initially, 39.2 mg of TiO_2_ (Aeroxid P25, Evonik Industries, Essen, Germany) and 0.8 mg of the given MWCNT were suspended in 100 mL of isopropanol (c = 400 mg·L^−1^) and ultrasonicated (Hielscher UP200S, Teltow, Germany) for 1 min. The well-mixed suspensions of the nanomaterials were filtered through the PVDF membranes in a batch-stirred membrane reactor (Millipore XFUF07601, Burlington, MA, USA) by using 0.3 MPa transmembrane pressure; then, the coated membranes were left to dry at room temperature overnight. The membranes were coated with 1 mg·cm^−2^ of nanocomposites.

### 2.2. Functionalization of MWCNT

To increase the hydrophilicity of the CNTs used for functionalization (Alfa Aesar, Waltham, MA, USA), we formed oxygen-containing functional groups (–OH and –COOH) on their surface. For this purpose, 250 mL of 15 M HNO_3_ and 10 M H_2_SO_4_/HNO_3_ mixture (at a *v*/*v* ratio of 3:1) solutions were prepared, and 1 g·L^−1^ CNTs were suspended in a round-bottomed flask [34]. The suspensions were allowed to react in an oil bath at 120 °C for 5 h in the presence of a water-cooled refluxer. The suspensions were stirred constantly (250 rpm) during the treatments. At the end of the reaction, the suspensions were sedimented by centrifugation (2000 rpm, 5 min). After discarding the supernatant, the CNTs were mechanically resuspended in ultrapure water (suspension concentration: 4 g·L^−1^) and sedimented again by centrifugation. This resuspension/centrifugation cycle was repeated eight times until reaching the neutral pH.

### 2.3. Filtration Experiments

The effects of the nanocomposite coatings of the membranes on the filtration performance were evaluated by filtering oil-in-water emulsions. Initially, crude oil (MOL Zrt, Algyő, Hungary) was vigorously blended with ultrapure water at 35,000 rpm for 1 min (Einhell TC-MG 135 E, Landau an der Isar, Germany). Subsequently, 20 mL of the thoroughly mixed oil dispersion was transferred into 480 mL of ultrapure water and homogenized for 10 min (200 W, 24 kHz, amplitude = 1, cycle = 100%) using ultrasonication (Hielscher UP200S, Teltow, Germany) to create stable oil-in-water emulsions. The as-prepared oil-in-water emulsions had the following properties: the chemical oxygen demand was 549 ± 12 mg·L^−1^, the turbidity was 280 ± 9 nephelometric turbidity units (NTU), the total oil and grease/total petroleum hydrocarbon (TOG/TPH) content was 108 ± 2.5 mg·L^−1^, and the pH value was 6.2 ± 0.1. The zeta potential (−43.3 ± 7.1 mV) and the oil droplet size distribution were described by dynamic light scattering measurements (Malvern ZetaSizer NanoZS, Malvern, UK; Figure S1), representing that the oil droplets had a diameter of 80–1300 nm with an average droplet size of 587 nm.

The filtration experiments were conducted in a pressure-driven (0.1 MPa) and batch-stirred (350 rpm) membrane reactor (Millipore XFUF07601, Burlington, MA, USA) with a feed volume of 250 mL. The experiments were continued until the volume reduction ratio (VRR) reached 5. The flux (J) was continuously measured by tracking the weight of the permeate and using the following equation:

(1) $$J=\frac{dV}{A_{m}\times dt}$$

where J is the flux, dV is the volume of the permeate (L), A_m_ is the active filtration area of the used membrane (m^2^), and dt is the time (h) at which the weight of the permeate was measured.

The fouling characteristics of the membranes were evaluated using a resistance-in-series model [35], which was calculated as follows. The membrane resistance was calculated using the following equation:

(2) $$R_{m}=\frac{∆p}{J_{w}\times {ƞ}_{w}}$$

where Δp is the applied transmembrane pressure (0.1 MPa), J_w_ is the water flux ensured by the clean membrane, and ƞ_w_ is the viscosity of the water at 25 °C (Pa·s).

Irreversible resistance is caused by the pollutants that remain on the membrane surface or within the membrane pores and cannot be removed from the membrane surface by rinsing with ultrapure water. Irreversible resistances were calculated using Equation (3):

(3) $$R_{irrev}=\frac{∆p}{J_{w}\times {ƞ}_{w}}-R_{m}$$

where Δp is the applied transmembrane pressure (0.1 MPa), J_w_ is the water flux after the oil filtration and the cleaning of the used membranes (via vigorous rinsing with ultrapure water), and ƞ_w_ is the viscosity of the water at 25 °C (Pa·s).

Reversible resistance refers to the washable pollutants deposited on the membrane surface during the filtration:

(4) $$R_{rev}=\frac{∆p}{J_{c}\times {ƞ}_{ww}}-R_{irrev}-R_{m}$$

where J_c_ is the steady-state flux at the end of the filtration (VRR = 5), and ƞ_ww_ is the viscosity of the wastewater (i.e., the used oil-in-water emulsion).

Total filtration resistances were calculated as the sum of the previous three resistance values:

(5) $$R_{total}=R_{m}+R_{rev}+R_{irrev}$$

The flux recovery ratio (FRR) provides information on how much of the initial water flux can be recovered by rinsing the membrane after the filtration of the oil emulsion, which can be calculated as:

(6) $$FRR=\frac{J_{wA}}{J_{w}}\times 100$$

where J_wA_ is the pure water flux after the filtration and cleaning of the membrane surface, and J_w_ is the pure water flux of the membrane before its utilization for the filtration of the oil emulsion.

The flux decay ratio (FDR) is used to compare the flux value at the end of the filtration of the oil emulsion (J_c_) to the clean water flux (J_w_; measured during the filtration of ultrapure water prior to the filtration of the oil emulsion):

(7) $$FDR=\frac{J_{w}-J_{c}}{J_{w}}\times 100$$

### 2.4. Characterization Methods

#### 2.4.1. Purification Efficiency

In addition to the filtration experiments, the purification effectiveness of the membrane filtration was also evaluated using chemical oxygen demand (COD) and turbidity assessments. The standard potassium dichromate oxidation method was used to measure the COD, which was conducted in standard test tubes (Hanna Instruments, Woonsocket, RI, USA) after digestion for 120 min at 150 °C. Turbidity values were measured using a nephelometric turbidity meter (Hach 2100 N, Loveland, CO, USA) and expressed in NTU. The purification efficiency (R) was calculated using Equation (8):

(8) $$R=\left(1-\frac{C}{C_{0}}\right)\times 100\%$$

#### 2.4.2. Membrane Surface Characterization

The zeta potentials of the membrane surfaces were determined using a streaming potential technique with an Anton Paar SurPASS 3 device (AntonPaar Gmbh, Graz, Austria) fitted with an adjustable gap cell. During the measurements, two pieces of the membranes (10 mm × 20 mm) were affixed with a double-sided adhesive tape. The measurements were conducted in the pH range of approximately 2–8 (the background electrolyte (KCl) concentration was 0.001 mol·L^−1^), which was adjusted by adding HCl and KOH solutions. The system was equipped with a pH electrode that continuously monitored the pH.

The wetting capabilities of the unmodified and modified membranes were evaluated by contact angle measurements (Dataphysics Contact Angle System OCA15Pro, Filderstadt, Germany). Ultrapure water (10 μL) was carefully added to the surfaces using a Hamilton pipette, and the contact angles between the membrane and ultrapure water droplets were quickly measured (within 1 s). Three repeated measurements were taken in all cases, and the average values were presented. Photos of underwater oil droplets on membrane surfaces were also captured with the same equipment.

#### 2.4.3. Gas Chromatography–Mass Spectrometry (GC-MS) Measurements of Crude Oils

The crude oil samples used to make the model emulsions were diluted 100-fold with hexane and analyzed using an Agilent 7890 B gas chromatograph coupled with an Agilent 5975C VL-MSD mass spectrometer. The mass spectrometer was set to an ion source temperature of 230 °C, quadrupole temperature of 150 °C, and an electron beam energy of 70 eV. Then, 1 μL of the sample was injected into the instrument using an Agilent 7683 B autosampler unit. The gas chromatograph was equipped with an HP-5ms Ultra Inert (30 m × 0.25 mm × 0.25 μm) column. Helium (purity = 6.0) was used as the carrier gas. Full-scan mode was used during the measurements. The initial oven temperature of 80 °C was maintained for 3 min and subsequently increased at a rate of 5 °C·min^−1^ to 325 °C. The final temperature was maintained for 3 min. The inlet was set to split mode with a split ratio of 19:1. The inlet temperature was 300 °C, and the column flow rate was 1.2 mL·min^−1^.

#### 2.4.4. Characterization of Functionalized Carbon Nanotubes

Fourier-transform infrared (FT-IR) spectra were recorded with a JASCO 4100 (Jasco, Tokyo, Japan) spectrometer in the range of 450–4500 cm^−1^ with 2 cm^−1^ spectral resolution using KBr pellets as a reference. The pellets were prepared by mixing 1.0–1.2 mg of CNT and 200 mg of KBr.

## 3. Results and Discussion

### 3.1. FT-IR Characterization of the MWCNTs

**Figure 1:**
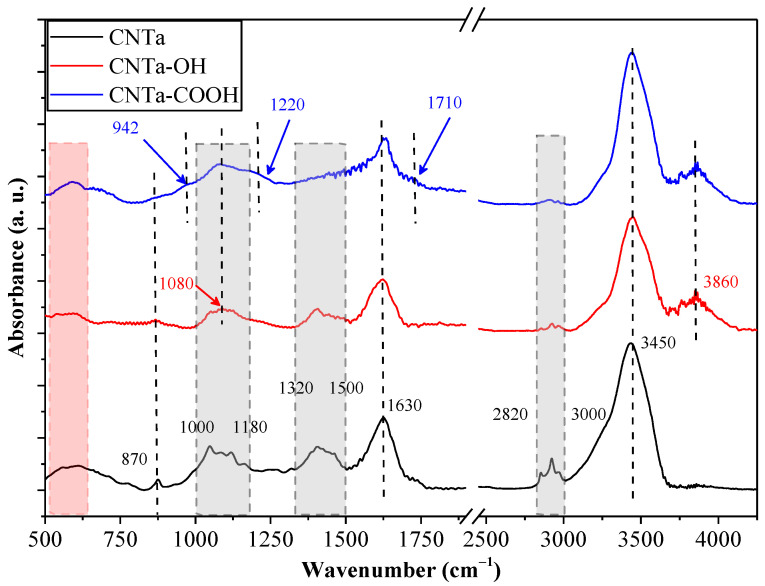
IR spectra of the CNT_a_ sample and its corresponding functionalized derivatives.

The IR spectra of the investigated samples revealed the specific surface features of the used nanotubes. CNT_a_ and CNT_b_ showed major differences before and after functionalization, while some similarities were also observed. Sample CNT_a_ (Figure 1) showed the basic characteristic vibration bands of CNTs at 870 cm^−1^ and 1630 cm^−1^, which were preserved following the functionalization [36]. The bands found between 1000–1180 cm^−1^ were attributed to different C–O vibrations, which may originate from various functional groups (e.g., ethers and esters) from the compounds used during their synthesis [37]. However, for bare CNT_a_, these were presumed to be dominantly non-polar functional groups, as suggested by the narrower O–H stretching band (3450 cm^−1^) and the absence of the band located at 3860 cm^−1^ [38]. The latter is a clear sign of the presence of H bonds. Nevertheless, the presence of the O–H stretching band signal is surprising in the bare CNT_a_ sample, suggesting water adsorption. The bands between 2820–3000 cm^−1^ indicate the presence of C_saturated_–H bonds [39]. The relative intensity of the signal decreased as the functionalization procedure occurred (CNT_a_–OH, CNT_a_–COOH). The complex overlapping IR signals between 1320–1500 cm^−1^ can be attributed to a wide range of functional groups and vibration modes, which are considered fingerprint regions for CNTs [37]. Therefore, the mentioned wavenumber interval is not considered in detail. However, it is clear that as the polarity of the surface increased, the intensity of the signal diminished. Following the functionalization with the OH groups, the signal in the 1000–1180 cm^−1^ region intensified, and a sharper peak appeared at 1080 cm^−1^, characteristic of the stretching vibrations of C–O bonds in alcohols, indicating the successful functionalization [40]. This signal was also present in sample CNT_a_–COOH. Moreover, following the anchoring of the –COOH groups, three new signals were observed: the first one at 942 cm^−1^ representing –OH wagging vibrations originating from the –COOH groups; the second one at 1220 cm^−1^ representing –OH stretching vibrations in the –COOH groups; the third one a shoulder located at 1710 cm^−1^ which was assigned to the C=O groups (in –COOH) [40]. It is important to mention that the presence of the previously mentioned signals does not exclude the possible existence of aldehydes and ketones on the surface.

**Figure 2:**
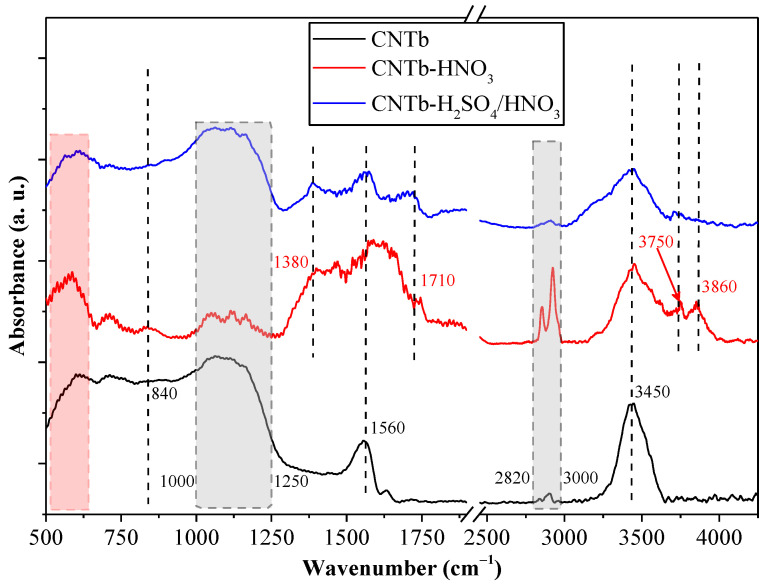
IR spectra of the CNT_b_ sample and the oxidation products obtained after acidic treatment.

For CNT_b_ (Figure 2), the basic spectral features were similar to those of CNT_a_. At 840 cm^−1^ and 1560 cm^−1^, the basic characteristic vibration bands of CNTs were found, corresponding to phonon coupling bands. An O–H stretching band (3450 cm^−1^) was also observed, while the 1000–1250 cm^−1^ C–O bond signals were also present (the upper limit of the interval was slightly higher).

The complex signal at 1320–1500 cm^−1^ was not explicitly present here, an issue that needs further investigation. Moreover, this signal was suppressed by the presence of bands related to NO_3_^−^ and N–O species [41] located at 1380 and 1560 cm^−1^ (samples CNT_b_–HNO_3_ and CNT_b_–H_2_SO_4_/HNO_3_), which could contribute to the polar surfaces of these samples. The shoulder located at 1710 cm^−1^ was also present in the oxidized samples, indicating the existence of surface anchored the –COOH groups. The successful completion of the oxidation process is also demonstrated by the appearance of the signals specific for H bonds originating from the polar groups at 3750 and 3860 cm^−1^. However, the efficiency of the oxidation of CNT_b_ cannot be determined from the IR spectra as the main spectral features are quite similar to those of CNT_b_–HNO_3_ and CNT_b_–H_2_SO_4_/HNO_3_. Unexpectedly, the intensity of the 2820–3000 cm^−1^ band was not in line with the observations made for the CNT_a_ sample. It should be noted that the 500–630 cm^−1^ region is another fingerprint region for carbon-based materials; thus, they were not considered in the analysis of either sample.

In summary, the presented IR spectra demonstrated the sufficient modification of both CNT_a_ and CNT_b_ surfaces, which proved the achievement of the desired functionalized CNT surfaces, which contained the polar and negative functional groups such as the –OH and –COOH groups.

### 3.2. Filtration Performance of the Membranes

First, the pure water fluxes were compared according to Table 2. As expected, the highest flux was measured for the uncoated PVDF membrane (5638 L·m^−2^·h^−1^), while the different coatings resulted in 22–36% lower water fluxes. Based on Table 2, TiO_2_ and the series of the three different TiO_2_/CNT_a_-coated membranes provided very similar (3645 ± 36 L·m^−2^·h^−1^) fluxes, while the other (b) series provided significantly higher water fluxes (4146–4419 L·m^−2^·h^−1^). Therefore, no significant effect of CNT functionalization can be observed by comparing the water fluxes of the two (a and b) series of the composite-coated membranes. However, significantly higher values can be observed for the functionalized membranes (TiO_2_/CNT_a_-OH, TiO_2_/CNT_a_-COOH, TiO_2_/CNT_b_-HNO_3_, and TiO_2_/CNT_b_-HNO_3__H_2_SO_4_) than for the basic CNTs (TiO_2_/CNT_a_ and TiO_2_/CNT_b_) by comparing the fluxes measured during the filtration of the emulsions at VRR = 1.5 (Table 2). This indicates a positive effect originating from the surface modifications of the CNTs.

Table 2 also summarizes the fluxes measured at the end of the filtration of the oil emulsions (at VRR = 5). First, it can be established that, due to the different nanomaterial coatings, significantly higher fluxes (37–104 L·m^−2^·h^−1^) were measured compared to the 24 L·m^−2^·h^−1^ flux value obtained for the uncoated membrane. The TiO_2_ coating resulted in a ~50% flux enhancement (37 L·m^−2^·h^−1^), and all the TiO_2_/CNT composite coatings were much more beneficial by providing up to four times higher fluxes (45–104 L·m^−2^·h^−1^) compared to the uncoated membrane. Still, it must be noted that, contrary to our expectations, the measured fluxes were very low in all cases compared to those presented in our previous study (up to 510 L·m^−2^·h^−1^) at the same 0.1 MPa transmembrane pressure [23].

**Figure 3:**
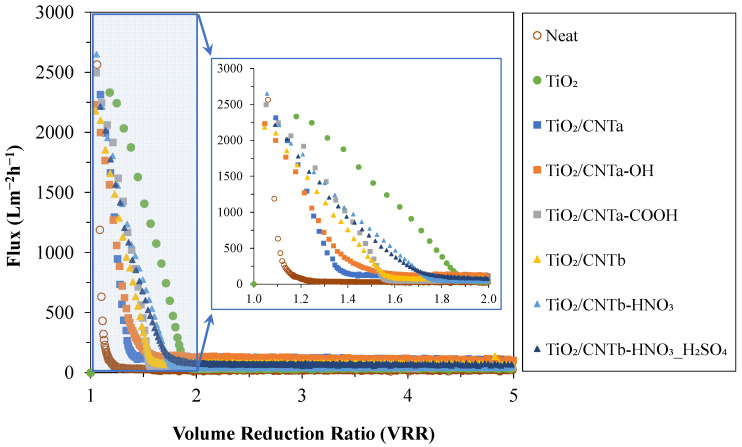
Flux values measured during the filtration of the oil emulsions (as a function of volume reduction ratio).

For further analysis of the results, Figure 3 shows the flux changes experienced during the filtration of the oil emulsion with the reference (neat) and nanocomposite-modified PVDF membranes up to a VRR of two and five.

Generally, a sharp decrease in the fluxes was observed for all membranes, and a steady-state effluent flux was achieved until reaching a VRR of two in all cases. Despite the initially high water flux (J_W_ = 5638 L·m^−2^·h^−1^), the reference (neat) PVDF membrane showed the quickest and most drastic flux decrease (the FDR was already ~90% at a VRR of 1.1). In contrast, the TiO_2_-modified membrane showed the slowest flux decrease (90% FDR was reached only at a VRR of ~1.8). For these two membranes, 30 and 1406 L·m^−2^·h^−1^ fluxes were measured at VRR = 1.5, respectively. The fluxes provided by all the TiO_2_/CNT nanocomposite-coated membranes were found to be between these two extremes at this initial filtration stage. These results partially contradict the findings of our previous study [23], in which the flux remained significantly high (510 L·m^−2^·h^−1^) until the end of the filtration (the FDR was only ~40% at a VRR of five) using the TiO_2_/CNT_2%_ nanocomposite-coated membrane. However, the fluxes provided by TiO_2_/CNT-coated membranes were up to ~2.8 and ~4 times higher (in both the present and the previous studies) compared to those provided by the TiO_2_-coated and the neat PVDF membranes, respectively. It is worth mentioning again that in the present study a different type of crude oil was used to prepare the emulsions. Therefore, in the next step, the compositions of the two oil samples were analyzed by GC-MS measurements to identify the possible compositional differences between the previously used oil (sample 1) and the oil used in this study (sample 2). The results are expected to clarify the reasons for the observed differences related to less effective flux enhancement of the TiO_2_/CNT composite coatings at the initial stage of the filtrations and the significantly lower flux values measured at the end of the filtration experiments (at VRR = 5).

### 3.3. Composition Analysis of Crude Oil Samples by Gas Chromatography

It is known that the stability of oil emulsions affects cake layer formation on membrane surfaces. Based on the literature, the stability of oil emulsions is significantly influenced by the oil composition (the polarity of individual hydrocarbons) and by the presence or absence of surfactants [42,43]. Since the heavy components of crude oils (e.g., asphaltenes and resins) are amphiphilic and thus can be regarded as natural surfactants [44,45], they might inhibit the coalescence of oil droplets and enhance emulsion stability [43,45,46].

**Figure 4:**
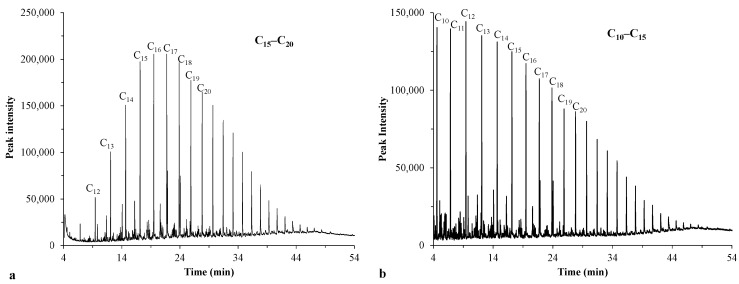
Chromatograms of crude oils: sample 1 (**a**) and sample 2 (**b**).

Figure 4a shows that the chromatogram of the previously used crude oil (sample 1) was dominated by medium-chain hydrocarbons (C15–C20). In contrast, the crude oil used in this study (sample 2) had a high proportion of shorter-chain hydrocarbons (C10–C15) (Figure 4b).

The individual components of the samples are listed in Tables S1 and S2. The measurements indicate that sample 1 contained several distinct hydrocarbon derivatives with polar molecular fractions due to heteroatoms or substituents, thus having a potential surfactant effect (for example, 1-iodoundecane, 2-amino-1-(o-methoxyphenyl)propane, 4-chloro-6-phenylpyrimidin-1-oxide, 6-amino-hex-2-en-1-ol, 1-iodo-hexadecane, 1-chloro-octadecane, and eicosanoic acid hexadecyl ester). According to the literature, such compounds can also contribute to the stability of the emulsions prepared from this oil (sample 1) by acting as natural emulsifiers [45,47,48]. This could account for the phenomenon observed in our prior study [23], in which the oil droplets were less likely to coalesce owing to the surfactant components, thus maintaining their increased flux. In contrast, the oil used in our current study (sample 2) had lower stability as an emulsion, indicating lower repulsion between the droplets. This could lead to more significant cake layer formation on the membrane surface during the filtration, which was reflected in the rapid flux reductions. Additionally, the results revealed that the crude oil used in this study (sample 2) contained a considerable number of organic compounds with π electrons (benzene, (1,1 dimethylpropyl); benzene, (1,2,3,5-tetramethyl); benzene, (1,2,3-trimethyl); naphtalene; naphtalene, 1,4,6-trimethyl, etc.). Moreover, it has been demonstrated that organic compounds with π electrons can form π–π bonds with the π electrons of CNT [49,50,51]. Consequently, the aromatic organic compounds of this oil could adhere to the surface of the CNT, which could contribute to the higher flux reductions observed for the TiO_2_/CNT nanocomposite-coated membranes in the initial filtration phase (in comparison with the flux curve of the membrane coated solely with TiO_2_).

### 3.4. Filtration Resistances

**Figure 5:**
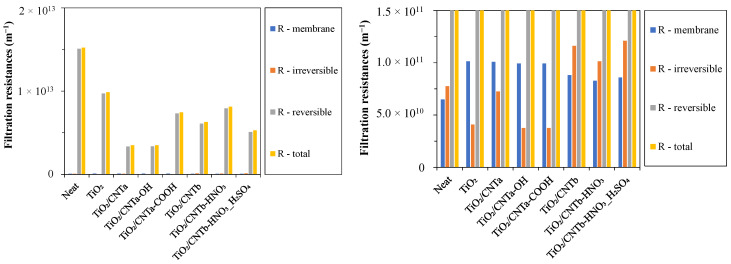
Filtration resistance values of the different membranes.

To gain a deeper understanding of the filtering performance of each membrane, we also calculated the different filtration resistances (Figure 5).

On the one hand, the obtained reversible resistances account for the largest fractions of the relatively high total filtration resistances for all the used membranes, indicating significant but easily removable hydrophobic cake layers on the surfaces that resulted in serious water barriers during filtrations. On the other hand, the membrane resistances and the irreversible resistances were very low compared to the total resistances. As expected, the membrane resistances of the coated membranes were slightly higher than that of the reference neat PVDF membrane in all cases. This is due to the additional resistance of the nanoparticle layer formed on the membrane surfaces. The very low irreversible resistances (much lower than in the previous study [23]), which originated from the irreversibly attached and pore-fouling small oil droplets, can be explained by the lower stability of the oil droplets and their higher ability to coalesce into larger droplets due to the lack of the natural emulsifier surfactant hydrocarbons in this type of oil. A similar finding was reported by Lu et al. [52]. They found that the surfactant in the oil emulsion significantly reduced its stability after adsorption onto the membrane, leading to the formation of larger oil droplets that could form a cake layer. This also explains the measured high total filtration resistances possibly caused by the more compact cake layer that formed due to the composition of the oil used in this study. The degree of irreversible resistance of the modified membranes can be divided into two groups. The irreversible resistances of the three TiO_2_/CNT_b_ nanocomposite-modified membranes were 2.5–3 times higher than those of the TiO_2_ and the three TiO_2_/CNT_a_-coated membranes. This can be attributed to the varying hydrophilicity and zeta potential of the composite membranes, which are discussed in the next chapter.

### 3.5. Comparison of the Surface Characteristics and the Filtration Properties

Table 3 lists the measured contact angles (which describe hydrophilicity) and zeta potentials of the membranes and, for further discussion, the calculated FRRs and FDRs. Additionally, photos of the water droplets on the different membranes are presented in Figure S2.

The neat membrane had the highest contact angle (46.6°) and, therefore, the lowest hydrophilicity within the series, with a slightly negative zeta potential (−11 mV). These characteristics resulted in a very low (45.5%) FRR and an exceptionally high (99.6%) FDR, in accordance with the previously presented relatively high irreversible resistance (compared to the TiO_2_ and the TiO_2_/CNT_a_-coated membranes) and extremely high total resistance. The contact angles of all TiO_2_/CNT-coated membranes represent hydrophilic surfaces. In the case of the TiO_2_/CNT_a_ series, the hydrophilicity apparently increased (as the contact angles decreased) via the CNT functionalization, which explains the increased fluxes (see the 117, 194, and 455 L·m^−2^·h^−1^ values of the TiO_2_/CNT_a_ series in Table 2) and the increased flux recovery ratios (Table 3). The underwater oil droplets (Figure S3) also proved the more oleophobic surfaces of the functionalized CNTs, especially for the CNT_a_-COOH sample (compared to CNT_a_). For the TiO_2_/CNT_b_ series, the effects of CNT functionalization could not be demonstrated by water contact angles, as they were 0° for all the TiO_2_/CNT_b_ coatings. Nevertheless, underwater oil droplets demonstrated the significantly more oleophobic surfaces of the CNT_b_-HNO_3_ and CNT_b_-HNO_3__H_2_SO_4_ nanotubes (compared to both the CNT_a_ and CNT_b_ nanotubes) in accordance with the very high fluxes (774 and 678 L·m^−2^·h^−1^, respectively) measured at the initial stages of the filtration (Table 2). However, it must be noted that all the TiO_2_/CNT_b_ coatings ensured similarly low FRRs (Table 3) despite the big differences in underwater oleophobicity, but the results can be explained by the zeta potentials.

The zeta potentials of the TiO_2_-coated and the three different TiO_2_/CNT_a_ nanocomposite-coated membranes (namely, TiO_2_/CNT_a_, TiO_2_/CNT_a_-OH, and TiO_2_/CNT_a_-COOH) were much more negative (−25, −22, and −40 mV, respectively), which proved to be beneficial since the zeta potential of the oil droplets was also strongly negative (−43 mV). This resulted in a greater electrostatic repulsion effect between the oil droplets and these membranes, thus decreasing the likelihood of the oil droplets sticking to the membrane surface or pores. Hence, these conditions contributed to the significantly higher FRRs (Table 3), the significantly lower irreversible resistances (Figure 5), and the relatively lower FDRs (Table 3) of these membranes compared to those of the neat membrane. In contrast, the zeta potentials of the TiO_2_/CNT_b_, TiO_2_/CNT_b_-HNO_3_, and TiO_2_/CNT_b_-HNO_3__H_2_SO_4_ membranes were −5, −4.5, and −6.5 mV, respectively. Therefore, despite the measured low contact angles (high hydrophilicity) and the oleophobic surfaces of these CNTs (Figure S3), the FRRs were similarly low (42.0–44.9%) as relates to the neat membrane (45.5%). At the same time, the irreversible resistances were by far the highest for these TiO_2_/CNT_b_ composite-coated membranes (Figure 5). These results demonstrate that the negative membrane surface becomes crucial for reducing membrane fouling at higher oil concentrations (at higher volume reduction ratios), and the absence of a negative membrane surface (negative zeta potential) cannot be compensated only by higher oleophobicity.

### 3.6. Oil Removal Efficiency

**Figure 6:**
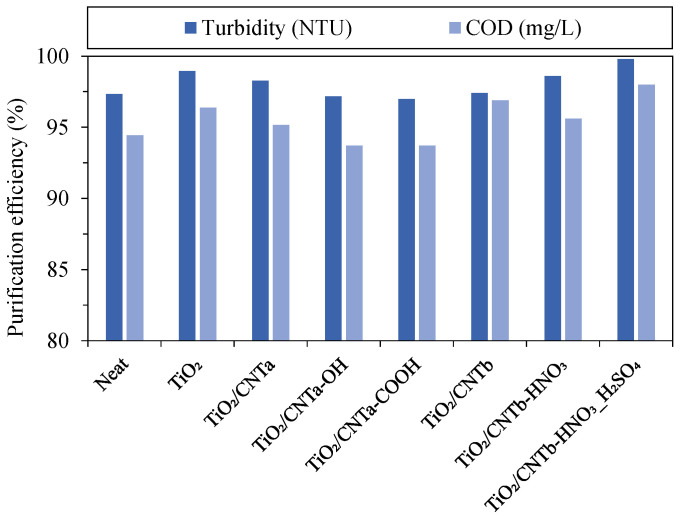
Purification efficiencies of the membranes (calculated from the turbidity and chemical oxygen demand values).

All membranes had satisfactory purification efficiency (Figure 6) with 97–99.8% (based on turbidity values), regardless of the membrane type. These numbers were slightly lower (93.7–98%) when the purification efficiency was evaluated in terms of COD. This discrepancy can be attributed to the fact that dissolved impurities that can pass through conventional microfilter membranes also contribute to the COD of the permeate. Nevertheless, comparable or superior results were obtained for the modified membranes compared to the reference membrane.

### 3.7. Comparative Discussion of the Results with Relevant Studies

To compare the results of this study with the literature, we compiled a table summarizing the main experimental conditions and results of some relevant studies (investigating TiO_2_- and/or CNT-coated membranes for the membrane filtration of oil emulsions; Table 4).

First, it must be noted that the direct comparisons of fluxes, rejections or FRR values do not necessarily lead to correct conclusions since even minor differences in the properties of the emulsions (composition, droplet size, zeta potential, etc.) and/or the properties of the composites (hydrophilicity, zeta potential, etc.) may result in significantly different filtration properties, as it has been demonstrated in the present study. What is clear, however, is that the highest flux values in the table (350–4777 L·m^−2^·h^−1^) are mostly related to those studies [28,29,55,58] in which the oil droplets were significantly bigger (1–10 µm) than in other studies in which nanoscaled oil droplets (d < 1 µm) were dispersed in the emulsions (like in the present study). The only exception to this general statement is the publication of Moslehyani et al. [32], in which the authors also developed an oxidized CNT-containing TiO_2_/CNT-composite-coated membrane and achieved a flux of 665 L·m^−2^·h^−1^. In line with that paper, our study also demonstrates that functionalized CNTs deserve close attention in the field of nanomaterial-modified (self-cleaning) membranes used for oil-in-water emulsion separation, as the demonstrated flux enhancements are prominent compared to the achievements of other relevant studies.

## 4. Conclusions

This study investigated the feasibility of using TiO_2_ and various TiO_2_/fCNT nanocomposite-coated PVDF microfilter membranes to purify oil-contaminated wastewater. For this purpose, commercially functionalized and self-functionalized CNTs were applied. The FT-IR results revealed that the surface of the CNTs was successfully modified with both the –OH and/or –COOH groups.

The filtration experiments proved that the functionalized membranes (TiO_2_/CNT_a_-OH, TiO_2_/CNT_a_-COOH, TiO_2_/CNT_b_-HNO_3_, and TiO_2_/CNT_b_-HNO_3__H_2_SO_4_) had significantly higher flux values at the initial stages (until VRR < 1.5) than the membranes coated with non-functionalized CNTs (TiO_2_/CNT_a_ and TiO_2_/CNT_b_), indicating the positive effect of the surface modification. Simple TiO_2_ coating also resulted in a notable (~50%) flux enhancement, but via the different TiO_2_/CNT coatings, much higher (~2–4 times higher) fluxes were achieved compared to the uncoated membrane (at VRR = 5).

The irreversible resistances of the membranes showed that the hydrophilicity and the zeta potential were simultaneously crucial for reducing the total membrane fouling. The zeta potentials of the TiO_2_-coated and TiO_2_/CNT_a_ nanocomposite-coated membranes (TiO_2_/CNT_a_, TiO_2_/CNT_a_-OH, and TiO_2_/CNT_a_-COOH) were significantly negative (−25, −22, and −40 mV, respectively). This was beneficial since the oil droplets of this study also had negative zeta potential (−43.3 mV) as usual, resulting in a significant electrostatic repulsion and reduced liability of the oil droplets to adhere onto the membrane surfaces or pores. In contrast, the zeta potentials of the TiO_2_/CNT_b_, TiO_2_/CNT_b_-HNO_3_, and TiO_2_/CNT_b_-HNO_3__H_2_SO_4_ membranes were only −5, −4.5, and −6.5 mV, respectively. Therefore, despite the measured low contact angles (high hydrophilicities), the irreversible resistances were by far the highest in these cases.

It was demonstrated that all membranes provided excellent purification efficiencies (97–99.8%; calculated from turbidity values). However, the fluxes decreased sharply (in line with the large total filtration resistances), reaching a steady-state effluent flux at VRR = 2. This decrease was the highest for the reference membrane (the FDR was ~90% at VRR = 1.1) and the lowest for the TiO_2_-modified membrane (the FDR became ~90% at a VRR of ~1.8). These findings partially contradict our recent study [27], where the flux remained high until the filtration ended (up to VRR = 5). However, a different crude oil was used in the present study, and gas chromatography measurements revealed that the crude oil used in the previous study contained hydrocarbon derivatives with polar molecular fractions due to heteroatoms or substituents that could act as natural emulsifiers. The oil used in this study contained negligible amounts of these emulsifying components; thus, their effect was less significant, making the oil droplets more prone to coalescence during filtration. These results led to a more significant cake layer on the membrane surface, a sharp decline in the flux, and much higher total filtration resistances compared to our previous study.

## Figures and Tables

**Table 1:** Characteristics of the carbon nanotubes used for modifying PVDF membranes.

Name of the MWCNT	Functionalization/ Modification	Manufacturer
CNT_a_	-	Nanografi
CNT_a_-OH	hydroxyl groups	Nanografi
CNT_a_-COOH	carboxyl groups	Nanografi
CNT_b_	-	Alfa Aesar
CNT_b_-HNO_3_	with 15 M HNO_3_	Alfa Aesar
CNT_b_-H_2_SO_4_/HNO_3_	with 10 M HNO_3_/H_2_SO_4_	Alfa Aesar

**Table 2:** Flux values measured during the filtration of ultrapure water and oil emulsions at VRR = 1.5 and 5.

Membranes	J_water_ (L·m^−2^·h^−1^)	J_emulsion_ at VRR = 1.5 (L·m^−2^·h^−1^)	J_emulsion_ at VRR = 5 (L·m^−2^·h^−1^)
Neat	5638	30	24
TiO_2_	3608	1406	37
TiO_2_/CNT_a_	3626	117	104
TiO_2_/CNT_a_-OH	3681	194	104
TiO_2_/CNT_a_-COOH	3678	455	49
TiO_2_/CNT_b_	4146	333	58
TiO_2_/CNT_b_-HNO_3_	4419	774	45
TiO_2_/CNT_b_-HNO_3__H_2_SO_4_	4255	678	69

**Table 3:** Membrane surface characteristics (hydrophilicity: described by contact angles; surface charges: described by zeta potential values), flux recovery ratios, and flux decay ratios.

Membranes	Contact Angles (°)	Zeta Potential at pH ~6 (mV)	FRR (%)	FDR (%)
Neat	46.6 ± 1.3	−11 ± 0.7	45.5	99.6
TiO_2_	0 ± 0	−25 ± 3.4	71.3	99.0
TiO_2_/CNT_a_	20.9 ± 1.9	−30 ± 5.3	58.2	97.1
TiO_2_/CNT_a_-OH	13.8 ± 0.8	−22 ± 4.3	72.6	97.2
TiO_2_/CNT_a_-COOH	4.4 ± 0.6	−40 ± 6.8	72.6	98.7
TiO_2_/CNT_b_	0 ± 0	−5.0 ± 2.5	43.1	98.6
TiO_2_/CNT_b_-HNO_3_	0 ± 0	−4.5 ± 1.3	44.9	99.0
TiO_2_/CNT_b_-HNO_3__H_2_SO_4_	0 ± 0	−6.5 ± 0.7	42.0	98.4

**Table 4:** Summary of the main experimental conditions and results of relevant studies.

Ref.	Membrane	Modifier Material	Type of Oil (Concentration)	Pressure	Effluent Flux (Lm^−2^h^−1^)	Rejection (%)	Fouling Indicators	Oil Droplet Size (µm)
[28]	CA	TiO_2_/SWCNT–COOH	hexadecane (1500 ppm)	0.1 MPa (vacuum)	~1500 (SWCNT) ~4777 (TiO_2_/SWCNT)	~98.7 (SWCNT) 99.5 (TiO_2_/SWCNT)	flux did not decreased after 10 cycle	1.38–1.91
[29]	PVDF	DTPA/MWCNT–COOH/TiO_2_-PVDF	cooking oil (–)	0.1 MPa (vacuum)	–(unmodifed) ~814 (modifed)	97.3 ± 0.6	flux slightly changed after 10 cycles	2.7–3.4
[32]	PVDF	MWCNTox-MWCNT	synt. refinery oil (100 ppm)	0.1 MPa	~500 (MWCNT) ~665 (ox-MWCNT)	~96 (MWCNT) ~99.8 (ox-MWCNT)	–	0.47
[53]	PVDF	HA-MWCNT	– (200 ppm)	0.3 Mpa	~30 (unmodifed) ~60 (modifed)	~60 (unmodifed) 88.7 (modifed)	(unmodifed) 82% FRR (modifed)	0.12
[54]	PVDF	ZrO_2_-MWCNT	diesel oil (–)	0.15 MPa	~50 (unmodifed) ~150 (modifed)	~90 (unmodifed) ~95 (modifed)	76.2% FRR (unmodifed) 90% FRR (modifed)	1.0–3.0
[55]	PVDF	DA/A-MWCNT	diesel oil (–)	0.09 MPa	–(unmodifed) ~886 (modifed)	–(unmodifed) 99 (modifed)	–(unmodifed) ~90% FRR (modifed)	1.0–10.0
[56]	PVDF	MWCNT-polypyrrole	crude oil (500 ppm)	0.2 MPa	~<30 (unmodifed) ~<100 (modifed)	90 (unmodifed) 99.5 (modifed)	~50% FRR (unmodifed) ~90% FRR (modifed)	0.4
[57]	PVDF	TiO_2_	cutting oil (250 ppm)	(vacuum)	28 (unmodifed) 72 (modifed)	~90 (unmodifed) ~97 (modifed)	–	1.08
[58]	Ceramic (Al_2_O_3_)	TiO_2_	hydraulic oil (–)	0.16 MPa	~245 (unmodifed) ~350 (modifed)	–	–	6
[59]	PVDF	D-K/TiO_2_	diesel oil (–)	0.09 (vacuum)	–(unmodifed) ~380 (modifed)	~99	almost recovers its initial flux	–
This study	PVDF	TiO_2_/ox-MWCNT	crude oil/400 ppm	0.1 MPa	24 (unmodifed) 104 (modifed)	>97	45.5% FRR(unmodifed) 72.6% FRR (modifed)	0.08–1.3

## Data Availability

Data are contained within the article.

## Supplementary Materials

The following supporting information can be downloaded at: https://www.mdpi.com/article/10.3390/membranes13100812/s1, Figure S1: Oil droplet size distribution of the used oil emulsion.; Figure S2: Water droplets on the different membrane surfaces and the measured contact angles (before the filtration of the oil emulsions). Figure S3: Underwater oil droplets on different membrane surfaces. Table S1: Unique components detected exclusively in sample 1 crude oil (analyzed by GC-MS); Table S2: Unique components detected exclusively in sample 2 crude oil (analyzed by GC-MS).

## References

[1] Putatunda S., Bhattacharya S., Sen D., Bhattacharjee C. (2018). A Review on the Application of Different Treatment Processes for Emulsified Oily Wastewater. Int. J. Environ. Sci. Technol..

[2] Yu L., Han M., He F. (2017). A Review of Treating Oily Wastewater. Arab. J. Chem..

[3] Abuhasel K., Kchaou M., Alquraish M., Munusamy Y., Jeng Y.T. (2021). Oily Wastewater Treatment: Overview of Conventional and Modern Methods, Challenges, and Future Opportunities. Water.

[4] Kundu P., Mishra I.M. (2018). Treatment and Reclamation of Hydrocarbon-Bearing Oily Wastewater as a Hazardous Pollutant by Different Processes and Technologies: A State-of-the-Art Review. Rev. Chem. Eng..

[5] Radelyuk I., Tussupova K., Zhapargazinova K. (2020). Impact of Oily Wastewater for Public Health in Rural Area: A Case Study of Kazakhstan.

[6] Shi G., Wu M., Zhong Q., Mu P., Li J. (2021). Superhydrophobic Waste Cardboard Aerogels as Effective and Reusable Oil Absorbents. Langmuir.

[7] al deen Atallah Ali D., Palaniandy P., Feroz S. (2019). Advanced Oxidation Processes (AOPs) to Treat the Petroleum Wastewater. Advances in Environmental Engineering and Green Technologies.

[8] Nascimbén Santos É., László Z., Hodúr C., Arthanareeswaran G., Veréb G. (2020). Photocatalytic Membrane Filtration and Its Advantages over Conventional Approaches in the Treatment of Oily Wastewater: A Review. Asia-Pac. J. Chem. Eng..

[9] Gan S., Li H., Zhu X., Liu X., Wei K., Zhu L., Wei B., Luo X., Zhang J., Xue Q. (2023). Constructing Scalable Membrane with Tunable Wettability by Electrolysis-Induced Interface pH for Oil–Water Separation. Adv. Funct. Mater..

[10] Otitoju T.A., Ahmad A.L., Ooi B.S. (2018). Recent Advances in Hydrophilic Modification and Performance of Polyethersulfone (PES) Membrane via Additive Blending. RSC Adv..

[11] Zhu Y., Xie W., Zhang F., Xing T., Jin J. (2017). Superhydrophilic In-Situ-Cross-Linked Zwitterionic Polyelectrolyte/PVDF-Blend Membrane for Highly Efficient Oil/Water Emulsion Separation. ACS Appl. Mater. Interfaces.

[12] Nascimben Santos E., Fazekas Á., Hodúr C., László Z., Beszédes S., Scheres Firak D., Gyulavári T., Hernádi K., Arthanareeswaran G., Veréb G. (2021). Statistical Analysis of Synthesis Parameters to Fabricate PVDF/PVP/TiO_2_ Membranes via Phase-Inversion with Enhanced Filtration Performance and Photocatalytic Properties. Polymers.

[13] Du Y., Li Y., Wu T. (2017). A Superhydrophilic and Underwater Superoleophobic Chitosan–TiO_2_ Composite Membrane for Fast Oil-in-Water Emulsion Separation. RSC Adv..

[14] Li H., Zhong Q., Sun Q., Xiang B., Li J. (2022). Upcycling Waste Pine Nut Shell Membrane for Highly Efficient Separation of Crude Oil-in-Water Emulsion. Langmuir.

[15] Esfahani M.R., Aktij S.A., Dabaghian Z., Firouzjaei M.D., Rahimpour A., Eke J., Escobar I.C., Abolhassani M., Greenlee L.F., Esfahani A.R. (2019). Nanocomposite Membranes for Water Separation and Purification: Fabrication, Modification, and Applications. Sep. Purif. Technol..

[16] Krishnan S.A.G., Sasikumar B., Arthanareeswaran G., László Z., Nascimben Santos E., Veréb G., Kertész S. (2022). Surface-Initiated Polymerization of PVDF Membrane Using Amine and Bismuth Tungstate (BWO) Modified MIL-100(Fe) Nanofillers for Pesticide Photodegradation. Chemosphere.

[17] Zhang J., Xue Q., Pan X., Jin Y., Lu W., Ding D., Guo Q. (2017). Graphene Oxide/Polyacrylonitrile Fiber Hierarchical-Structured Membrane for Ultra-Fast Microfiltration of Oil-Water Emulsion. Chem. Eng. J..

[18] Jianqiang Z., Hui L., Peizhi L., Xilu L., Shaopeng G., Xiao C., Lei Z., Baojun W., Qingzhong X. (2022). Recyclable Superhydrophilic Meshes with Scalable and Robust Coating for Separating Oily Wastewater. Appl. Surf. Sci..

[19] Chen P.-C., Xu Z.-K. (2013). Mineral-Coated Polymer Membranes with Superhydrophilicity and Underwater Superoleophobicity for Effective Oil/Water Separation. Sci. Rep..

[20] Kim J., Van der Bruggen B. (2010). The Use of Nanoparticles in Polymeric and Ceramic Membrane Structures: Review of Manufacturing Procedures and Performance Improvement for Water Treatment. Environ. Pollut..

[21] Mahdi N., Kumar P., Goswami A., Perdicakis B., Shankar K., Sadrzadeh M. (2019). Robust Polymer Nanocomposite Membranes Incorporating Discrete TiO_2_ Nanotubes for Water Treatment. Nanomaterials.

[22] de Oliveira C.P.M., Fernandes Farah I., Koch K., Drewes J.E., Viana M.M., Amaral M.C.S. (2022). TiO_2_-Graphene Oxide Nanocomposite Membranes: A Review. Sep. Purif. Technol..

[23] Fekete L., Fazekas Á.F., Hodúr C., László Z., Ágoston Á., Janovák L., Gyulavári T., Pap Z., Hernadi K., Veréb G. (2023). Outstanding Separation Performance of Oil-in-Water Emulsions with TiO_2_/CNT Nanocomposite-Modified PVDF Membranes. Membranes.

[24] Ma D., Zou X., Zhao Z., Zhou J., Li S., Yin H., Wang J. (2022). Hydrophilic PAA-g-MWCNT/TiO_2_@PES Nano-Matrix Composite Membranes: Anti-Fouling, Antibacterial and Photocatalytic. Eur. Polym. J..

[25] Shooshtari M., Salehi A. (2022). An Electronic Nose Based on Carbon Nanotube -Titanium Dioxide Hybrid Nanostructures for Detection and Discrimination of Volatile Organic Compounds. Sens. Actuators B Chem..

[26] Veréb G., Kálmán V., Gyulavári T., Kertész S., Beszédes S., Kovács G., Hernádi K., Pap Z., Hodúr C., László Z. (2018). Advantages of TiO_2_/Carbon Nanotube Modified Photocatalytic Membranes in the Purification of Oil-in-Water Emulsions. Water Supply.

[27] Veréb G., Kassai P., Nascimben Santos E., Arthanareeswaran G., Hodúr C., László Z. (2020). Intensification of the Ultrafiltration of Real Oil-Contaminated (Produced) Water with Pre-Ozonation and/or with TiO_2_, TiO_2_/CNT Nanomaterial-Coated Membrane Surfaces. Environ. Sci. Pollut. Res..

[28] Sun Y., Zhao R., Wang Q., Zheng Y., Li G., Sun D., Wu T., Li Y. (2020). Superwetting TiO_2_-Decorated Single-Walled Carbon Nanotube Composite Membrane for Highly Efficient Oil-in-Water Emulsion Separation. Korean J. Chem. Eng..

[29] Venkatesh K., Arthanareeswaran G., Chandra Bose A., Suresh Kumar P., Kweon J. (2021). Diethylenetriaminepentaacetic Acid-Functionalized Multi-Walled Carbon Nanotubes/Titanium Oxide-PVDF Nanofiber Membrane for Effective Separation of Oil/Water Emulsion. Sep. Purif. Technol..

[30] Tian S., He Y., Zhang L., Li S., Bai Y., Wang Y., Wu J., Yu J., Guo X. (2023). CNTs/TiO_2_- Loaded Carbonized Nanofibrous Membrane with Two-Type Self-Cleaning Performance for High Efficiency Oily Wastewater Remediation. Colloids Surf. A Physicochem. Eng. Asp..

[31] Esfahani M.R., Tyler J.L., Stretz H.A., Wells M.J.M. (2015). Effects of a Dual Nanofiller, Nano-TiO_2_ and MWCNT, for Polysulfone-Based Nanocomposite Membranes for Water Purification. Desalination.

[32] Moslehyani A., Ismail A.F., Othman M.H.D., Matsuura T. (2015). Design and Performance Study of Hybrid Photocatalytic Reactor-PVDF/MWCNT Nanocomposite Membrane System for Treatment of Petroleum Refinery Wastewater. Desalination.

[33] Nascimben Santos E., Ágoston Á., Kertész S., Hodúr C., László Z., Pap Z., Kása Z., Alapi T., Krishnan S.A.G., Arthanareeswaran G. (2020). Investigation of the Applicability of TiO_2_, BiVO_4_, and WO_3_ Nanomaterials for Advanced Photocatalytic Membranes Used for Oil-in-water Emulsion Separation. Asia-Pac. J. Chem. Eng..

[34] Sezer N., Koç M. (2019). Oxidative Acid Treatment of Carbon Nanotubes. Surf. Interfaces.

[35] Kovács I., Veréb G., Kertész S., Hodúr C., László Z. (2017). Fouling Mitigation and Cleanability of TiO_2_ Photocatalyst-Modified PVDF Membranes during Ultrafiltration of Model Oily Wastewater with Different Salt Contents. Environ. Sci. Pollut. Res..

[36] Kim J.Y. (2009). Carbon Nanotube-Reinforced Thermotropic Liquid Crystal Polymer Nanocomposites. Materials.

[37] Kim U.J., Furtado C.A., Liu X., Chen G., Eklund P.C. (2005). Raman and IR Spectroscopy of Chemically Processed Single-Walled Carbon Nanotubes. J. Am. Chem. Soc..

[38] Efimov A.M., Pogareva V.G., Shashkin A.V. (2003). Water-Related Bands in the IR Absorption Spectra of Silicate Glasses. J. Non-Cryst. Solids.

[39] Hastings S.H., Watson A.T., Williams R.B., Anderson J.A. (1952). Determination of Hydrocarbon Functional Groups by Infrared Spectroscopy. Anal. Chem..

[40] de Menezes B.R.C., Ferreira F.V., Silva B.C., Simonetti E.A.N., Bastos T.M., Cividanes L.S., Thim G.P. (2018). Effects of Octadecylamine Functionalization of Carbon Nanotubes on Dispersion, Polarity, and Mechanical Properties of CNT/HDPE Nanocomposites. J. Mater. Sci..

[41] Abdolmohammad-Zadeh H., Tavarid K., Talleb Z. (2012). Determination of Iodate in Food, Environmental, and Biological Samples after Solid-Phase Extraction with Ni-Al-Zr Ternary Layered Double Hydroxide as a Nanosorbent. Sci. World J..

[42] Zhu X., Dudchenko A., Gu X., Jassby D. (2017). Surfactant-Stabilized Oil Separation from Water Using Ultrafiltration and Nanofiltration. J. Membr. Sci..

[43] Kokal S. (2005). Crude-Oil Emulsions: A State-Of-The-Art Review. SPE Prod. Facil..

[44] Martínez-Palou R., de Lourdes Mosqueira M., Zapata-Rendón B., Mar-Juárez E., Bernal-Huicochea C., de la Cruz Clavel-López J., Aburto J. (2011). Transportation of Heavy and Extra-Heavy Crude Oil by Pipeline: A Review. J. Pet. Sci. Eng..

[45] Cao G., Du T., Bai Y., Yang T., Zuo J. (2021). Effects of Surfactant Molecular Structure on the Stability of Water in Oil Emulsion. J. Pet. Sci. Eng..

[46] Varjani S.J. (2017). Microbial Degradation of Petroleum Hydrocarbons. Bioresour. Technol..

[47] Koshlaf E., Ball A.S. (2017). Soil Bioremediation Approaches for Petroleum Hydrocarbon Polluted Environments. AIMS Microbiol..

[48] Patil A., Arnesen K., Holte A., Farooq U., Brunsvik A., Størseth T., Johansen S.T. (2021). Crude Oil Characterization with a New Dynamic Emulsion Stability Technique. Fuel.

[49] Zhao J., Lu J.P., Han J., Yang C.-K. (2003). Noncovalent Functionalization of Carbon Nanotubes by Aromatic Organic Molecules. Appl. Phys. Lett..

[50] Liu T., Chen S., Liu H. (2015). Oil Adsorption and Reuse Performance of Multi-Walled Carbon Nanotubes. Procedia Eng..

[51] Kayvani Fard A., Mckay G., Manawi Y., Malaibari Z., Hussien M.A. (2016). Outstanding Adsorption Performance of High Aspect Ratio and Super-Hydrophobic Carbon Nanotubes for Oil Removal. Chemosphere.

[52] Lu D., Zhang T., Ma J. (2015). Ceramic Membrane Fouling during Ultrafiltration of Oil/Water Emulsions: Roles Played by Stabilization Surfactants of Oil Droplets. Environ. Sci. Technol..

[53] Abdulazeez I., Salhi B., Elsharif A.M., Ahmad M.S., Baig N., Abdelnaby M.M. (2023). Hemin-Modified Multi-Walled Carbon Nanotube-Incorporated PVDF Membranes: Computational and Experimental Studies on Oil–Water Emulsion Separations. Molecules.

[54] Yang X., He Y., Zeng G., Zhan Y., Pan Y., Shi H., Chen Q. (2016). Novel Hydrophilic PVDF Ultrafiltration Membranes Based on a ZrO_2_–Multiwalled Carbon Nanotube Hybrid for Oil/Water Separation. J. Mater. Sci..

[55] Yang X., He Y., Zeng G., Chen X., Shi H., Qing D., Li F., Chen Q. (2017). Bio-Inspired Method for Preparation of Multiwall Carbon Nanotubes Decorated Superhydrophilic Poly(Vinylidene Fluoride) Membrane for Oil/Water Emulsion Separation. Chem. Eng. J..

[56] Hudaib B., Abu-Zurayk R., Waleed H., Ibrahim A.A. (2022). Fabrication of a Novel (PVDF/MWCNT/Polypyrrole) Antifouling High Flux Ultrafiltration Membrane for Crude Oil Wastewater Treatment. Membranes.

[57] Ong C.S., Lau W.J., Goh P.S., Ng B.C., Ismail A.F. (2015). Preparation and Characterization of PVDF–PVP–TiO_2_composite Hollow Fiber Membranes for Oily Wastewater Treatment Using Submerged Membrane System. Desalination Water Treat..

[58] Chang Q., Zhou J., Wang Y., Liang J., Zhang X., Cerneaux S., Wang X., Zhu Z., Dong Y. (2014). Application of Ceramic Microfiltration Membrane Modified by Nano-TiO_2_ Coating in Separation of a Stable Oil-in-Water Emulsion. J. Membr. Sci..

[59] Shi H., He Y., Pan Y., Di H., Zeng G., Zhang L., Zhang C. (2016). A Modified Mussel-Inspired Method to Fabricate TiO_2_ Decorated Superhydrophilic PVDF Membrane for Oil/Water Separation. J. Membr. Sci..

