# Supplementary material for: Effects of Different TiO2/CNT Coatings of PVDF Membranes on the Filtration of Oil-Contaminated Wastewaters
Source: Membranes (Basel). 2023 Sep 27;13(10):812. doi: 10.3390/membranes13100812 (PMC10608089; doi:10.3390/membranes13100812)
Supplement: Supplementary file 1 [file membranes-13-00812-s001.zip › membranes-2614247-supplementary.pdf]

membranes

MDPI

Supplementary material

Effects of Different TiO_2/CNT Coatings of PVDF Membranes on the Filtration of Oil-Contaminated Wastewaters

Ákos Ferenc Fazekas [1,2], Tamás Gyulavári [3], Zsolt Pap [3,4,5], Attila Bodor [6,7], Krisztián Laczi [6], Katalin Perei [6], Erzsébet Illés [8], Zsuzsanna László [1,*] and Gábor Veréb [1,*]

[1] Department of Biosystem Engineering, Faculty of Engineering, University of Szeged, Moszkvai Blvd. 9., H-6725 Szeged, Hungary; fazekas@mk.u-szeged.hu
[2] Doctoral School of Environmental Sciences, University of Szeged, Rerrich Béla Sq. 1, H-6720 Szeged, Hungary
[3] Department of Applied and Environmental Chemistry, Institute of Chemistry, University of Szeged, Rerrich Béla Sq. 1, H-6720 Szeged, Hungary; gyulavarit@chem.u-szeged.hu (T.G.); pzsolt@chem.u-szeged.hu (Z.P.)
[4] Centre of Nanostructured Materials and Bio-Nano Interfaces, Institute for Interdisciplinary, Research on Bio-Nano-Sciences, Treboniu Laurian 42, RO-400271 Cluj-Napoca, Romania
[5] STAR-UBB Institute, Mihail Kogălniceanu 1, RO-400084 Cluj-Napoca, Romania
[6] Department of Biotechnology, Institute of Biology, University of Szeged, Közép Alley 52, H-6726 Szeged, Hungary; bodor.attila@brc.hu (A.B.); laczi.krisztian@brc.hu (K.L.); perei@bio.u-szeged.hu (K.P.)
[7] Institute of Biophysics, Biological Research Centre, Hungarian Research Network, Temesvári Blvd. 62, H-6726 Szeged, Hungary
[8] Department of Food Engineering, University of Szeged, Mars Sq. 7, H-6724 Szeged, Hungary; erzsebet.illes@mk.u-szeged.hu
* Correspondence: zsizsu@mk.u-szeged.hu (Z.L.); verebg@mk.u-szeged.hu (G.V.)

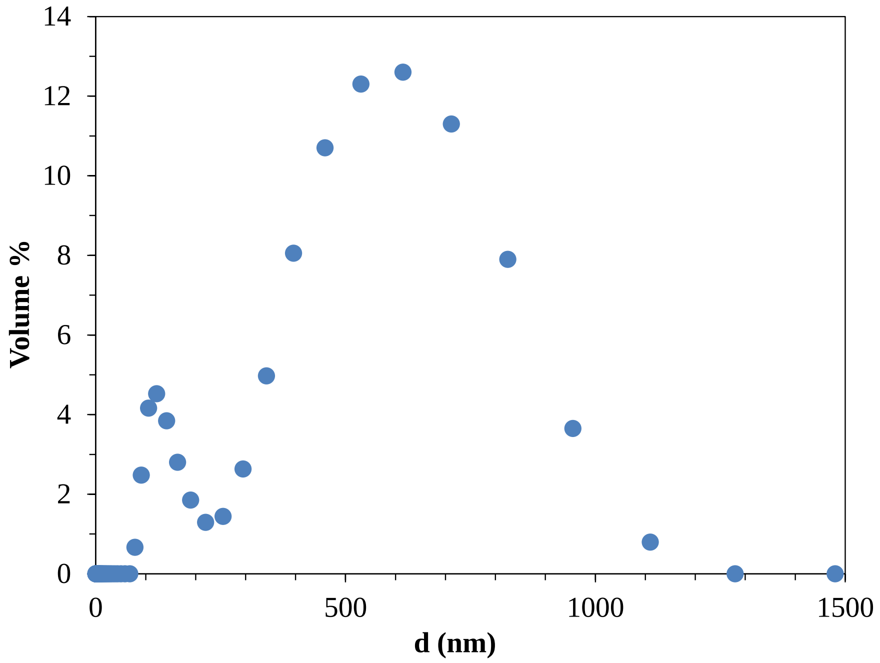

Figure S1. Oil droplet size distribution of the used oil emulsion.

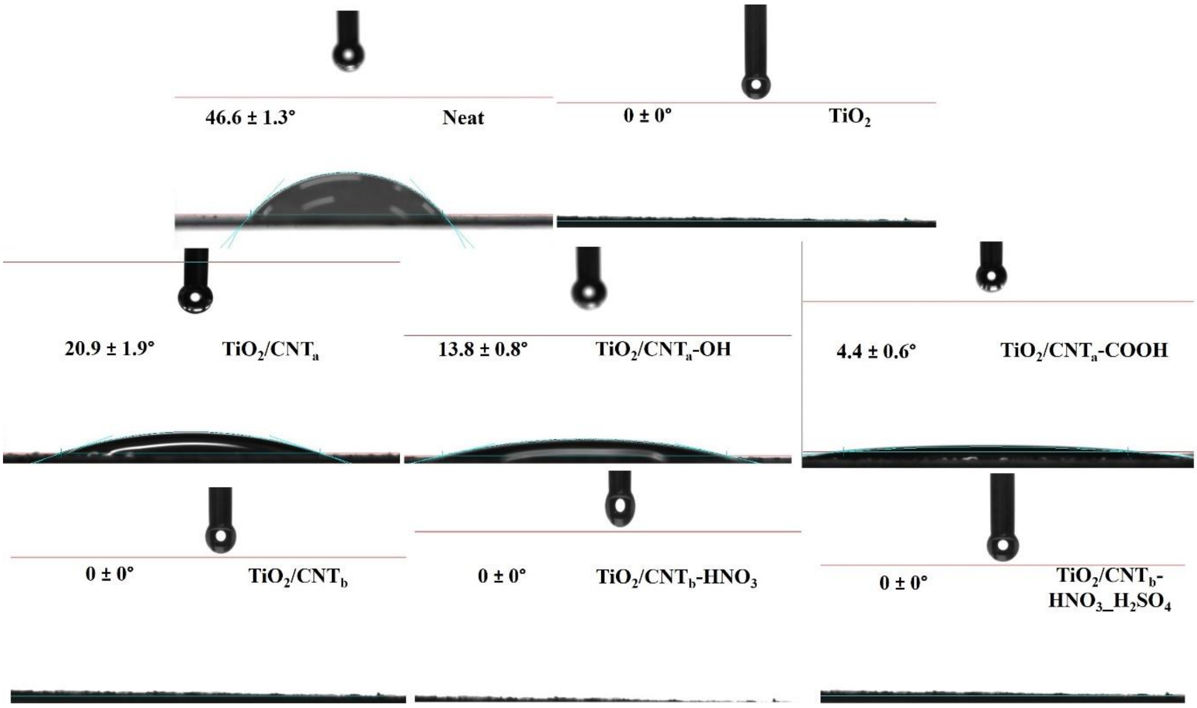

Figure S2. Water droplets on the different membrane surfaces and the measured contact angles (before the filtration of the oil emulsions).

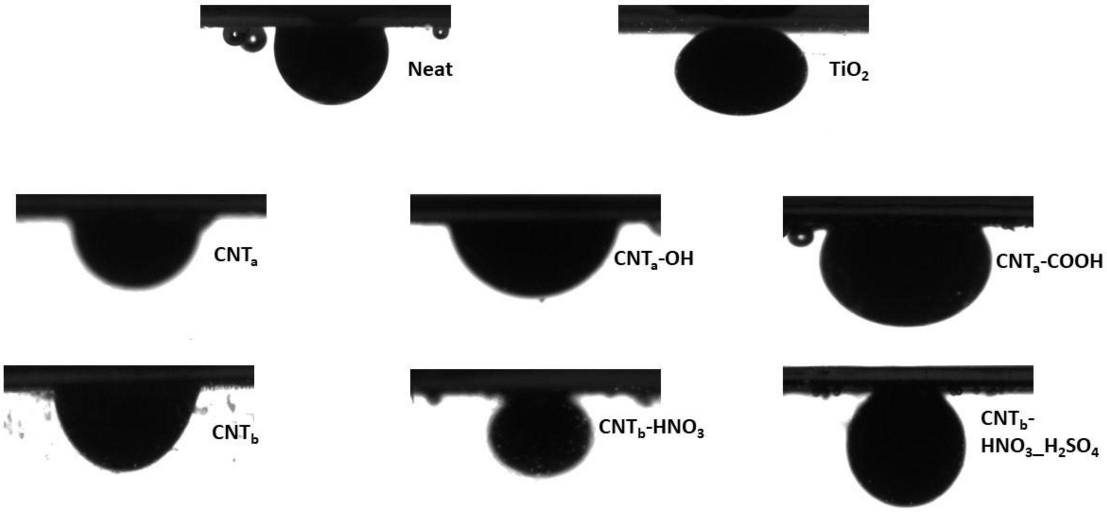

Figure S3. Underwater oil droplets on different membrane surfaces.

Table S1. Unique components detected exclusively in sample 1 crude oil (analyzed by GC-MS).

17-Pentatriacontene
1-Iodoundecane
1-Nonadecene
2-Amino-1-(o-methoxyphenyl)propane
2-Bromo dodecane
2-Furanmethanamine, tetrahydro-

3,3,7,7-Tetramethyl-5-oxa-1,9-diazabicyclo[4.3.0]non-8-ene
3,5-Dimethyldodecane
4-Chloro-6-phenylpyrimidine-1-oxid
6-Amino-hex-2-en-1-ol
Anthracene
Anthracene, 1-methyl-
Benzene, 1,3-bis(3-phenoxyphenoxy)
Cyclobutene-3,4-dione, 1-dimethyla
Cyclohexane, (1-hexyltetradecyl)-
Cyclohexane, 1,1'-(1,3-propanediyl)bis-
Cyclohexane, decyl-
Dibenzothiophene, 3-methyl-
Dodecane, 2,6,10-trimethyl-
Dodecane, 2-methyl-8-propyl-
Eicosanoic acid, hexadecyl ester
Heneicosane
Heptadecane, 3-methyl-
Heptadecane, 9-octyl-
Hexadecane, 1-iodo-
Hexadecane, 4-methyl-
Naphthalene, 1-(2-propenyl)-
Naphthalene, 1,3-dimethyl-
Naphthalene, 2,3,6-trimethyl-
Nonadecane, 2,6,10,14,18-pentamethyl-
Nonadecane, 4-methyl-
Octacosyl acetate
Octadecane, 1-chloro-
Octadecane, 1-iodo-
Octane, 2,6-dimethyl-
Pentacosane
Pentadecane, 4-methyl-
Pentadecane, 8-hexyl-
Phenanthrene, 2,5-dimethyl-
Tetradecane, 3-methyl-
Tetradecane, 6,9-dimethyl-
Triacontane
Tridecane, 1-iodo-

35

36

Table S2. Unique components detected exclusively in sample 2 crude oil (analyzed by GC-MS).

1,1'-Biphenyl, 4-methyl-
10-Methylnonadecane
1-Docosene
1H-Indene, 2,3-dihydro-4,7-dimethyl-
3-Hexene, 3-ethyl-2,5-dimethyl-
Benzene, (1,1-dimethylpropyl)-
Benzene, 1,2,3,5-tetramethyl-
Benzene, 1,2,3-trimethyl-
Benzene, 1-chlorodifluoromethoxy-4-nitro-
Benzene, 1-methyl-3-propyl-
Benzene, 1-methyl-4-(1-methylethyl)-
Benzene, 2-ethyl-1,4-dimethyl-
Cyclohexane, 1-ethyl-2-methyl-, cis-
Cyclohexane, butyl-
Cyclohexane, pentyl-
Cyclohexane, undecyl-
Cyclopentane, (1-methylethyl)-
Cyclopentane, 1-butyl-2-pentyl-
Cyclopropane, 1-methyl-1-(2-methylpropyl)-2-nonyl-
Decane
Decane, 2-methyl-
Decane, 3,7-dimethyl-
Decane, 3-methyl-
Decane, 4-methyl-
Decane, 5-methyl-
Docosane
Docosane, 11-butyl-
Dodecane, 2-methyl-6-propyl-
Dodecane, 4-methyl-
Heptacosane, 1-chloro-
Heptadecane, 2,6,10,14-tetramethyl
Hexacosane
Hexadecane, 2-methyl-
Naphthalene
Naphthalene, 1,4,6-trimethyl-
Naphthalene, 1,4-dimethyl-
Nonane, 5-butyl-
Pentadecane, 2,6,10-trimethyl-
Pentadecane, 3-methyl-
Pentadecane, 7-methyl-
Tetradecane
Tetradecane, 4,11-dimethyl-
Tridecane, 3-methyl-
Tridecane, 5-methyl-
Tridecane, 7-methyl-
Undecane
Undecane, 2-methyl-
Undecane, 3-methyl-
Undecane, 4-methyl-
Undecane, 6-methyl-
